# Porcine Cytomegalovirus/Porcine Roseolovirus, Previously Transmitted During Xenotransplantation, Does Not Infect Human 293T and Mouse Cells with Impaired Antiviral Defense

**DOI:** 10.3390/v18010021

**Published:** 2025-12-23

**Authors:** Hina Jhelum, Reinhold Schäfer, Benedikt B. Kaufer, Joachim Denner

**Affiliations:** 1 Institute of Virology, Free University Berlin, 14163 Berlin, Germany; hina.jhelum@fu-berlin.de (H.J.); benedikt.kaufer@fu-berlin.de (B.B.K.); 2 Comprehensive Cancer Center, Charité Universitätsmedizin Berlin, 10117 Berlin, Germany; reinhold.schaefer@charite.de

**Keywords:** porcine cytomegalovirus/porcine roseolovirus (PCMV/PRV), herpesviruses, xenotransplantation, infection assays

## Abstract

Porcine cytomegalovirus, more accurately classified as porcine roseolovirus (PCMV/PRV), was shown to be pathogenic in the context of xenotransplantation. Transmission of PCMV/PRV to non-human primates receiving hearts or kidneys from virus-positive pigs significantly reduced the survival time of the recipients. PCMV/PRV was also transmitted to the first human recipient of a pig heart transplant and contributed to the patient’s death. Although PCMV/PRV is highly prevalent in all pig breeds and wild boars, including slaughterhouse pigs, no infections or diseases have been reported in healthy, ill, or immunocompromised humans, suggesting that this virus is not zoonotic and should therefore be classified as xenozoonotic. This indicates that this virus is not zoonotic and must be classified as xenozoonotic. Moreover, it remains unclear whether PCMV/PRV is capable of infecting human cells in vitro. To address this question, human 293T cells resistant to hygromycin were co-cultured with porcine fallopian tube (PFT) cells producing PCMV/PRV. After hygromycin selection, the remaining human cells showed no evidence of infection. Because herpesviruses are generally considered to be species-specific—a notion that has been shown to be not entirely correct—it was also investigated whether PCMV/PRV can infect mouse cells using the same approach. Similarly, no infection was observed. Since the target cells employed in both assays had a reduced capacity to resist viral infection, the findings strongly suggest that PCMV/PRV is unable to infect human or mouse cells, which are equipped with functional antiviral mechanisms. This is supported by findings from the patient who received the first pig heart transplantation.

## 1. Introduction

Xenotransplantation using genetically modified pigs offers a promising solution to the shortage of allogeneic donor organs and is steadily advancing toward clinical application. Long-term survival has been achieved in non-human primates transplanted with hearts and kidneys from genetically modified pigs, and the first transplantations of pig hearts, kidneys, and livers into human patients and brain-dead individuals have been reported [1]. However, xenotransplantation carries the potential risk of transmitting porcine microorganisms, particularly viruses, to the recipient, and preventing such transmission is of critical importance [2,3].

The transmission of a porcine herpesvirus, porcine cytomegalovirus, more accurately classified as porcine roseolovirus (PCMV/PRV) and officially designated Suid herpesvirus 2 (SuHV-2) [4], to the first human recipient of a pig heart transplant, where it contributed to the patient’s death [5], highlights the critical importance of ensuring viral safety in xenotransplantation. In addition to viral transmission, a combination of complications—including drug effects, immunosuppression, surgical stress, and the patient’s poor pre-transplant condition—contributed to the patient’s death.

PCMV/PRV is widespread in pigs, being detected in both wild boars [6,7] and slaughterhouse pigs [8,9], with nearly all slaughterhouse animals carrying the virus. Despite this broad distribution, no cases of PCMV/PRV transmission to healthy, ill, or immunocompromised humans have been reported, indicating that the virus is not zoonotic. This contrasts with hepatitis E virus genotype 3 (HEV3), which is zoonotic and causes chronic HEV infections, hepatitis, and manifestations of extrahepatic diseases in infected individuals [10,11]. The risks associated with acquiring HEV3 include occupational exposure to infected pigs and consumption of undercooked pork or pork products. Pig caretakers and swine veterinarians are at an increased risk of HEV infection [12].

At present, it remains unclear whether PCMV/PRV can infect human cells. One study reported infection of human fibroblasts [13], whereas another showed that co-cultivation of PCMV-infected pig macrophages with two human cell lines (293 and Raji) did not result in virus transmission [14]. In our own preliminary infection studies, using PBMCs from PCMV/PRV-positive pigs and human 293 cells, no evidence of infection was observed.

Therefore, additional experiments are needed to determine whether PCMV/PRV can infect human cells. It is also of interest to assess whether mouse cells are susceptible to PCMV/PRV. Although herpesviruses are generally considered species-specific, this notion has been shown to be not entirely correct [15]. For this reason, we used hygromycin-resistant human and mouse cells, co-incubated them with porcine fallopian tube (PFT) cells producing PCMV/PRV, and subsequently eliminated the pig cells by adding hygromycin selection medium. Whereas uninfected PFT cells could be infected with PCMV/PRV produced by infected PFT cells, either through co-cultivation or exposure to cell-free virus-containing supernatant, human and mouse cells could not be infected.

## 2. Materials and Methods

### 2.1. PCMV/PRV-Producing PFT Cells

PFT cells infected with PCMV/PRV were kindly supplied by Dr. N.J. Mueller, University Hospital Zurich, University of Zurich, Switzerland. PFT cells producing PCMV/PRV were derived from the inner lining of the pig oviduct, also evident by the presence of Barr bodies in these cells. They were adherent and had both epithelial- and fibroblast-like morphology. These cells were characterized by increased nuclear–cytoplasmic ratio as well as increased size and number of nucleoli [16,17,18,19]. A total of 17 of 24 RNA viruses and 8 of 9 DNA viruses replicate in this cell line [20]. They were cultured in Dulbecco’s modified Eagle medium (DMEM) (Gibco, Thermo Fisher Scientific, Waltham, MA, USA) supplemented with 10% heat-inactivated fetal bovine serum (FBS) (PAN Biotech, Aidenbach, Germany), 2mM L-Glutamine (PAN Biotech, Aidenbach, Germany), 1x non-essential amino acids (NEAs) (Biochrom GmbH, Berlin, Germany), 100 µg/mL streptomycin, and 60 µg/mL penicillin (PAN Biotech, Aidenbach, Germany) in an incubator (Heracell, Thermo Fisher Scientific, Waltham, MA, USA) with 5% CO_2_ at 37 °C. Upon confluency, cells were detached using Accutase (Invitrogen, Waltham, MA, USA) and split for expansion.

### 2.2. Hygromycin-Resistant Human Cells

Human embryonic kidney 293T cell line harboring a construct expressing p15E-NHR-His, a part of the transmembrane envelope protein of the porcine endogenous retrovirus (PERV) together with the hygromycin resistance gene (293Thyg) [21], were cultured in DMEM with 10% heat-inactivated FBS, 100 µg/mL streptomycin, and 60 µg/mL penicillin in 5% CO_2_ incubator at 37 °C.

### 2.3. Hygromycin-Resistant Mouse Cells

Embryo fibroblasts from double-stranded RNA-dependent protein kinase (PKR) knockout mice were treated according to the 3T3 protocol [22]. These 3T3-like cells are immortal. In these cells the induction of type I interferon (IFN) as well as the activation of NF-kB by poly(I)-poly(C) (pIC) were strongly impaired [23]. The cells were transfected with a plasmid, pY3, having a hygromycin resistance gene. One clone, MI4Y-3/8, was cultured in low-glucose DMEM GlutaMAX (Gibco, Thermo Fisher Scientific, Waltham, MA, USA) supplemented with 10% heat-inactivated FBS and 1% penicillin/streptomycin (PAN Biotech, Aidenbach, Germany) in 5% CO_2_ incubator at 37 °C.

### 2.4. Transfection of PFT Cells

PFT cells were seeded at a cell density of 2 × 10^5^ per well in a 6-well plate in 2 mL of serum-free DMEM media a day before transfection. Then, 1 µg of pVITRO-eGFP plasmid [24] DNA was mixed with sterile water up to 20 µL in a microfuge tube. The tube was gently flicked for mixing. Afterward, 200 µL of Opti-MEM (Gibco, Thermo Fisher Scientific, Waltham, MA, USA) was added to the tube containing the plasmid and vortexed mildly, followed by a short spin. Subsequently, 8 µL of polyethylenimine (PEI; Sigma-Aldrich, St. Louis, MO, USA) was added to the tube and vortexed mildly again, followed by incubation at room temperature for 20 min. The transfection mixture was added to the cells overnight. The next day, the medium was removed and replaced with fresh serum containing DMEM. After 2 days, 400 µg/mL hygromycin was added. Cells were monitored for the expression of eGFP under a fluorescence microscope (Zeiss Axio, Oberkochen, Germany). Upon enrichment of transfected cells and removal of non-transfected ones, they were expanded for subsequent experiments.

### 2.5. Co-Cultivation and Selection

**Figure 1:**
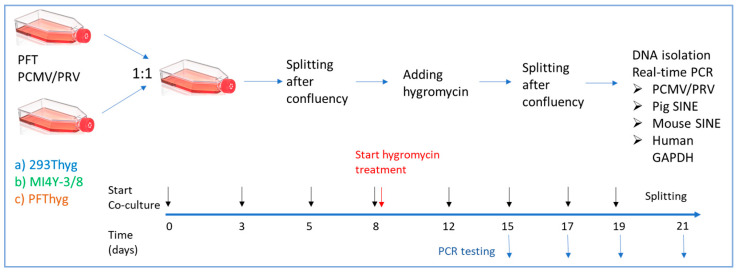
Schematic overview of the experiments co-cultivating PCMV/PRV-producing PFT cells with 293Thyg, MI4Y-3/8 and uninfected PFThyg cells. The timeline refers to co-cultivation with human 293hyg cells; for the other cell lines, small variations in the number of days were applied. Black arrows denote the time points of splitting, and blue arrows denote the time points of PCR testing.

PFT cells producing PCMV/PRV were co-cultured with 293Thyg cells and MI4Y-3/8 cells (Figure 1), respectively, at a cell density of 0.5 × 10^6^ cells for each cell line/5 mL in a T25 flask (Sarstedt AG & Co. KG, Numbrecht, Germany) at 37 °C in a 5% CO_2_ incubator. At day 3, when cells became confluent, they were split into two T25 flasks, followed by another round of splitting the cells upon reaching confluency. Post attachment of the cells to the flask surface, 400 µg/mL hygromycin was added to the flask for selection of 293Thyg and MI4Y-3/8 cells. After 2 days, cells were split again and maintained in 400 µg/mL hygromycin (Carl Roth, Karlsruhe, Germany) containing complete DMEM until confluency. Co-cultured cells with 293Thyg became confluent in 2 days, whereas cells co-cultured with MI4Y-3/8 became confluent in 4 days. Cells were washed twice with PBS to remove any remaining PFT-producing PCMV/PRV cells. Cells were detached using Accutase (Invitrogen, Waltham, MA, USA) and processed for DNA isolation and real-time PCR to test for PCMV/PRV, human glyceraldehyde-3-phosphate-dehydrogenase (hGAPDH), as well as short interspersed nuclear element (SINE) sequences.

As a positive control for the assay, PFT cells transfected with the pVITRO-eGFP vector were also co-cultured with PFT cells producing PCMV/PRV. After 5 days, 400 µg/mL hygromycin was added to the flask for selection of PFT-pVITRO-eGFP cells. They were monitored under a fluorescence microscope and real-time PCR for PCMV/PRV.

### 2.6. Cell-Free Infection

Supernatant from PFT cells producing PCMV/PRV was centrifuged twice (300 rpm, 10 min, and 500 rpm, 15 min, at room temperature) to remove any remaining cells. It was filtered using a 0.2 µm filter (Millipore, Burlington, MA, USA) and added repeatedly to the uninfected PFT cells after splitting.

### 2.7. Virus Pelleting

Supernatant from PFT cells producing PCMV/PRV was collected in a similar way as described in Section 2.6. and ultracentrifuged at 30,000 rpm, 4 °C for 1.5 h using SW32 Ti-rotor (Beckmann Coulter, Brea, CA, USA). The pellet was resuspended in 200 µL PBS, and DNA was isolated and analyzed by PCR.

### 2.8. DNA Isolation

DNA was isolated from the cell lines and the virus pellet using DNeasy Blood & Tissue kit (Qiagen, Hilden, Germany) following the manufacturer’s instructions. DNA was quantified using a NanoDrop ND-1000 (Thermo Fisher Scientific Inc., Worcester, MA, USA).

### 2.9. Real-Time PCR for PCMV/PRV, GAPDH, and SINE

Real-time PCRs using specific primers and probes (Table 1) were performed with DNA isolated from the respective cell lines to test for PCMV/PRV infection. They were performed using a SensiFAST Probe No-ROX kit (Meridian Bioscience, Cincinnati, OH, USA) in a reaction volume of 16 µL plus 4 µL (100 ng) of DNA template. All real-time PCRs were carried out as duplex PCRs, simultaneously testing the virus gene of interest and hGAPDH/murine SINE (SINE B1)/porcine SINE (PRE-1)/porcine GAPDH (pGAPDH) as the internal control for each sample. Real-time PCR reactions were carried out with a qTOWER^3^ G qPCR cycler (Analytik Jena, Jena, Germany).

For PCMV/PRV, hGAPDH, pGAPDH, and murine SINE, the temperature–time profile consisted of an initial inactivation step at 50 °C for 2 min, followed by denaturation at 95 °C for 10 min, and 45 cycles of denaturation at 95 °C for 15 s, and annealing/extension at 60 °C for 1 min. For porcine SINE, the temperature–time profile consisted of an initial inactivation step at 95 °C for 5 min, followed by 45 cycles of denaturation at 95 °C for 15 s, annealing at 60 °C for 30 s, and extension at 72 °C for 30 s.

### 2.10. Microscopy

A total of 1 × 10^6^ cells were seeded in a T25 cell culture flask (Sarstedt AG & Co. KG, Numbrecht, Germany) and imaged using a Zeiss Axio microscope using ZEN 2.3 software (Oberkochen, Germany) with a 10× objective. Brightfield and fluorescent images were acquired. Images were processed using ImageJ [25].

**Table 1:** Primers and probes.

Primers and Probes Used for Real-Time PCR	Sequence 5′-3′	Nucleotide Position	Accession Number	Reference
PCMV/PRV fw PCMV/PRV rev PCMV/PRV probe	ACTTCGTCGCAGCTCATCTGA GTTCTGGGATTCCGAGGTTG 6FAM-CAGGGCGGCGGTCGAGCTC-BHQ1	45,206–45,226 45,268–45,249 45,247–45,229	AF268039	Mueller et al., 2002 [26]
hGAPDH fw hGAPDH rev hGAPDH probe	GGCGATGCTGGCGCTGAGTAC TGGTTCACACCCATGACGA HEX-CTTCACCACCATGGAGAAGGCTGGG-BHQ1	3568–3587 3803–3783 3655–3678	AF261085	Behrendt et al., 2009 [27]
pGAPDH fw pGAPDH rev pGAPDH probe	GATCGAGTTGGGGCTGTGACT ACATGGCCTCCAAGGAGTAAGA HEX-CCACCAACCCCAGCAAGAG-BHQ	1083–1104 1188–1168 1114–1137	NM_001206359.1	Duvigneau et al., 2005 [28]
PRE-1 fw PRE-1 rev PRE-1 probe	GACTAGGAACCATGAGGTTGCG AGCCTACACCACAGCCACAG FAM-TTTGATCCCTGGCCTTGCTCAGTGG-BHQ1	37–58 61–85 151–170	Y00104	Walker et al., 2003 [29]
SINE B1 fw SINE B1 rev SINE B1 probe	TGGCGCACGCCTTTAATC TGGCCTCGAACTCAGAATCC 6FAM-ACTCGGGAGGCAGAGG-BHQ1	n.a.	n.a.	Gualtieri et al., 2013 [30]
PERV-C fw PERV-C rev	CTGACCTGGATTAGAACTGG ATGTTAGAGGATGGTCCTGG	6606–6625 6867–6886	AM229312	Takeuchi et al., 1998 [31]

n.a., not applicable.

## 3. Results

### 3.1. Characterization of PCMV/PRV Used in the Infection Studies

PFT cells and PFT cells producing PCMV/PRV are well characterized; both were found to be positive for porcine endogenous retrovirus (PERV), and the latter were positive for PCMV/PRV [16,17,18,19,20]. The cell line was originally derived from the inner lining of the pig oviduct, also known as porcine fallopian tubes (PFTs) or uterine tubes [16]. This line was susceptible to 17 of 24 porcine RNA viruses and 8 of 9 porcine DNA viruses, among them was PCMV/PRV [20]. PCMV/PRV-infected cells showed a cytomegalic cytopathogenic effect and intranuclear inclusion [17]. Recently, virus particles were isolated from a culture supernatant and characterized by electron microscopy [32]. The virus released from PFT cells was shown to infect uninfected PFT cells [18,19,32], as well as porcine aortic endothelial cells (PAECs) in vitro, leading to the activation of these cells, as demonstrated by increased expression of porcine tissue factor [33].

**Figure 2:**
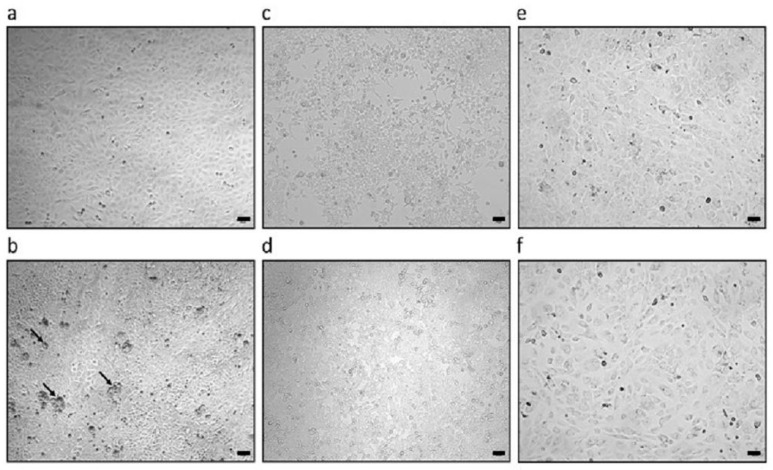
Morphology of the used cell lines before and after co-cultivation. (**a**) Uninfected PFT cells; (**b**) PFT cells producing PCMV/PRV; (**c**) untreated 293T cells; (**d**) 293 cells after co-culture with PFT cells producing PCMV/PRV and selection; (**e**) untreated MI4Y-3/8 cells; (**f**) MI4Y-3/8 cells after co-culture with PFT cells producing PCMV/PRV and selection. Scale bar indicates 100 µm, and magnification is 10×. Large round PCMV/PRV-producing cells are indicated by arrows.

The PFT cells used here were PCMV/PRV-negative, and the PFT cells producing PCMV/PRV (Figure 2) were positive, as confirmed by real-time PCR analysis (Table 2). Both cell lines were like all porcine cells positive for porcine endogenous retroviruses PERV-A and PERV-B, but they were negative for PERV-C (Table 2).

In the PCMV/PRV-producing cell line, large round cells were visible (Figure 2b), which should represent PCMV/PRV-producing cells, as has been shown previously by Fischer et al. [32] using immunological methods.

To determine whether PCMV/PRV is released into the supernatant of PCMV/PRV-positive PFT cells, DNA was repeatedly isolated directly from the culture supernatant and analyzed by real-time PCR for the presence of viral DNA. In all cases, PCMV/PRV was not detected. Therefore, the supernatant was first centrifuged to remove cells, filtrated, and then ultracentrifuged to pellet viral particles. PCMV/PRV was subsequently detected in the virus pellet (Table 3). A total of 50 ng DNA was extracted from the virus pellet; the measured 10^2.4^ viral copies corresponded to 5 mL of supernatant.

### 3.2. PCMV/PRV Infection of Hygromycin-Resistant PFT Cells

**Figure 3:**
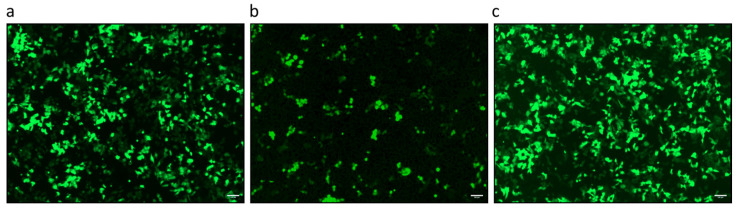
Morphology of the PFThyg cells before, during, and after co-cultivation with PFT cells producing PCMV/PRV. (**a**) uninfected PFThyg cells; (**b**) PFThyg cells co-cultured with PFT cells producing PCMV/PRV; (**c**) PFThyg cells after selection by hygromycin. Scale bar is 100 µm, and magnification is 10×.

In order to obtain hygromycin-resistant PFT cells, PFT cells were transfected with pVITRO-eGFP, and selection was performed with hygromycin. The selected cells showed expression of GFP in all cells and hygromycin resistance (PFThyg) (Figure 3). PFThyg cells were co-cultivated with PFT cells producing PCMV/PRV, as shown in Figure 1. Upon selection with hygromycin, virus-producing PFT cells were eliminated, followed by screening for PCMV/PRV and porcine SINE by real-time PCR (Table 4). The presence of the SINE sequence indicates the porcine origin of the cells. The PFThyg cells were PCMV/PRV-positive, demonstrating proof of concept for PCMV/PRV infection experiments.

In both the PCMV/PRV-producing PFT cells as well as the PFT cells freshly infected with PCMV/PRV, we found, by real-time PCR, 10^4^ copies per 100 ng DNA. Since 100 ng DNA corresponds to 1.7 × 10^4^ cells (assuming 6 pg DNA per cell), approximately 58.8% of the cells were infected, assuming that each infected cell carries only one viral copy.

### 3.3. Failure to Infect Human Cells

Human 293T cells were made resistant to hygromycin (293Thyg) by transfecting them with a construct expressing, in addition to hygromycin, a fragment of the transmembrane envelope protein p15E of PERV used for other purposes [21]. These cells were co-cultured with PFT cells producing PCMV/PRV by splitting the cells in between upon confluency, followed by screening for PCMV/PRV by real-time PCR. A schematic representation of the co-culture experimental set-up is shown in Figure 1. At day 21, both PCR assays—the one detecting PCMV and the one detecting pig SINE—were negative, indicating that no pig cells remained and that human cells were not infected (Table 4). To detect virus infection, we used the detection of viral DNA by real-time PCR. Since this method is highly sensitive (10 copies/100 ng DNA [9]) and the results were negative at different time points after starting selection, there was no need to screen for viral transcripts or proteins. To note, brightfield microscopy images of 293Thyg cells after co-cultivation and selection (Figure 2) demonstrated the absence of morphologically PFT-PCMV/PRV-specific cells.

### 3.4. Failure to Infect Mouse Cells

Mouse cells derived from RNA-dependent protein kinase (PKR) knockout mice, which were made hygromycin-resistant [23] (Figure 2), were co-cultivated with PFT cells producing PCMV/PRV in a similar manner as with 293Thyg cells (Figure 1). After removal of the pig cells by adding selection medium containing 400 µg/mL hygromycin, the mouse cells were tested for PCMV/PRV and pig SINE, where both PCRs were negative (Table 4). As an additional control, murine SINE B1 was detected in the treated cells.

### 3.5. Infection of PFT Cells by Supernatant from PCMV/PRV-Producing PFT Cells

To assess whether PFT cells can be infected by cell-free PCMV/PRV, the supernatant from PCMV/PRV-positive PFT cells was centrifuged to remove residual cells, filtered through a 0.2 µm filter, and repeatedly added to uninfected PFT cells at each passage together with fresh medium. This procedure was performed every four days over a period of 25 days. At each passage, the cells were washed with PBS, and part of them was analyzed by real-time PCR for the presence of PCMV/PRV. Although PCMV/PRV was not detected directly in the supernatant, likely due to a low viral copy number, it was present in the pelleted virus fraction (Table 3) and was capable of infecting PFT cells (Table 5). This indicates that PCMV/PRV can establish cell-free infection, although the infection rate is lower compared with the rate after co-culture (15% vs. 58.8%).

## 4. Discussion

PCMV/PRV has been shown to reduce the survival time of xenotransplants when transmitted by an organ from virus-infected donor pigs. This was shown in the case of pig thymokidneys in baboons [33], pig kidneys in baboons [35], pig kidneys in cynomolgus monkeys [36], and pig hearts in baboons [37]. Notably, PCMV/PRV was transmitted to the first human recipient of a pig heart and contributed to the patient’s death [5].

In baboon recipients, PCMV-expressing cells were found in all organs by immunohistochemistry, indicating the presence of cells expressing viral proteins [38]. High levels of PCMV/PRV DNA, detected by PCR in all baboon organs, confirmed this finding [38]. The concurrent identification of porcine SINE sequences in the same samples [34] suggests that these corresponded to disseminated pig cells.

Since humans have not been reported to be infected with PCMV/PRV under natural conditions, and the virus causes disease only in the context of xenotransplantation, it is not considered zoonotic but should instead be classified as xenozoonotic [39]. This indicates that humans face no occupational or dietary exposure risks. In baboons, in the context of xenotransplantation, the virus induced consumptive coagulopathy and thrombocytopenia [33]. Analyzing the cytokine levels in the blood, an increase in IL-6 and TNFα in baboons with PCMV/PRV-positive hearts was observed [36]. Furthermore, high levels of tissue plasminogen activator (tPA) and plasminogen activator inhibitor 1 (tPA-PAI-1) complexes were found, suggesting a complete loss of the pro-fibrinolytic properties of the endothelial cells [36].

Only two studies have addressed the question of whether PCMV/PRV can infect human cells. In one, human nasal fibroblasts were inoculated with supernatants from persistently infected porcine turbinate cells (PT-K75 cells), and infection was demonstrated by a cytopathic effect by 7 days post-infection, and reverse-transcriptase polymerase chain reaction sequencing identified PCMV/PRV RNA polymerase transcripts in these infected cells [13]. However, this study lacked critical controls, and the possibility of transmission of virus-producing pig cells could not be excluded. Surprisingly, a monoclonal antibody to human CMV (HCMV) glycoprotein B was used to detect PCMV/PRV. In our studies no cross-reaction between PCMV/PRV and HCMV was observed using the recombinant gB protein of PCMV/PRV [40]. A second study used persistently infected primary porcine alveolar macrophages (PAMs) as a source of PCMV/PRV, which were co-cultivated, for up to 15 passages, with human B (RAJI) and 293 cells. No PCMV/PRV infection was observed by PCR [14]. Consistently, our preliminary infection experiments using PBMCs from PCMV/PRV-positive pigs and human 293 cells also revealed no infection.

In addition to our finding that PCMV/PRV does not infect human cells with impaired antiviral defense, and the report by Tucker et al. [14] showing that PCMV/PRV does not infect human cells in vitro, strong in vivo evidence also indicates that PCMV/PRV does not infect non-human primate or human cells. First, in baboons transplanted with PCMV/PRV-positive pig hearts, PCMV/PRV DNA was detected by PCR in all analyzed organs [36]. Only very few cells expressing PCMV/PRV proteins were identified in baboon organs by immunohistochemistry [37]. Porcine SINE sequences were detected by PCR in these organs [38], indicating the presence of disseminated pig cells. Second, single-cell RNA sequencing demonstrated that PCMV/PRV transcripts were present only in pig cells, and not in baboon cells from these same animals (Reichart D. et al., Department of Medicine I, University Hospital, LMU Munich, personal communication). Third, in the patient who received the first pig heart transplant, detection of PCMV/PRV was consistent with the presence of porcine cell microchimerism in the examined organs [41]. Although PCMV/PRV transcription was identified in the transplanted porcine heart—indicating active viral replication—recipient tissues, including the liver, spleen, and kidney, tested negative for PCMV/PRV transcription [41].

Herpesviruses were traditionally considered strictly species-specific and incapable of crossing species barriers. However, this view has changed with accumulating evidence demonstrating transspecies transmission in several herpesviruses [42]. For example, baboon cytomegalovirus (BaCMV) has been shown to infect human cells in vitro and was detected in human recipients of baboon liver transplants [43,44]. Similarly, human cytomegalovirus (HCMV) has been reported to infect pig cells [45]. In contrast, PCMV/PRV appears to remain strictly species-specific. Previous work has demonstrated that PCMV/PRV failed to replicate in rabbits, mice, hamsters, chick embryos, or cattle [46], reinforcing its species specificity. Since cell-free transmission was less efficient than cell-to-cell spread, PCMV/PRV relies heavily on direct membrane contact, a feature that would further limit its ability to jump species in vivo.

We deliberately selected cell lines with impaired antiviral defense mechanisms for our infection experiments. In 293T cells, a downregulation of several genes involved in viral sensing and restriction has been reported, including apolipoprotein B mRNA editing enzyme catalytic subunit 3G (APOBEC3G), stimulator of interferon genes (STING), Janus kinase 3 (JAK3), IFI16, guanylate-binding protein 5 (GBP5), bone marrow stromal cell antigen 2 (BST2), and MX2 [47,48]. Moreover, 293 cells are deficient in Toll-like receptors (TLRs) and Nod2 [49]. These cells are profoundly defective in cytosolic DNA sensing and interferon induction, and multiple HEK293 cell lines—regardless of their source—naturally lack detectable cGAS or STING protein expression [50,51].

The functions of these factors are well characterized. APOBEC3G functions as a cytidine deaminase that introduces cytidine-to-uridine mutations, thereby compromising viral coding sequences and replication capacity. STING detects viral nucleic acids and triggers the production of interferons α and β. JAK3, a member of the Janus family of tyrosine kinases associated with cytokine receptors, mediates downstream signaling upon cytokine binding; its inhibition is known to exert immunosuppressive effects, for instance, in organ transplantation. The interferon-inducible protein IFI16 contributes to viral DNA sensing [52], whereas GBP5, a dynamin-like GTPase, promotes NLRP3 inflammasome activation and plays an important role in innate immunity. BST2, better known as tetherin, prevents the release of budding viral particles from the cell surface. The interferon-induced GTP-binding protein Mx2 displays antiviral activity against HIV-1 and herpesviruses [53]. Finally, cyclic GMP–AMP synthase (cGAS) acts as a cytosolic DNA sensor that activates type I interferon responses; notably, cGAS-deficient mice are more susceptible to lethal infections caused by both DNA and RNA viruses [54].

Mouse target cells were derived from mice lacking functional double-stranded RNA-dependent protein kinase (PKR) [23]. PKR has been implicated in the induction of interferons (IFNs) and in the antiviral response. In these mice, the antiviral activity induced by IFN-γ and poly(I:C) (pIC) was reduced; however, the induction of type I IFN genes by pIC and viral infection remained unimpaired. In contrast, embryonic fibroblasts derived from PKR knockout mice exhibited a pronounced impairment in both type I IFN induction and NF-κB activation following pIC stimulation.

Most interferon-stimulated genes (ISGs) encode signaling molecules that enhance cellular responsiveness to pathogens and propagate immune signals from localized sites of infection. PKR serves as an effector molecule that mediates antiviral responses through the phosphorylation of protein substrates, thereby activating signaling pathways involved in homeostasis, immune regulation, and—under sustained activation—apoptosis [55]. IFN signaling induces PKR expression; however, PKR remains inactive until activated by binding to double-stranded RNA (dsRNA) [56,57]. Interaction with dsRNA triggers PKR dimerization and autophosphorylation, leading to kinase activation. Activated PKR then catalyzes serine/threonine phosphorylation of various substrates, most notably the α-subunit of eukaryotic translation initiation factor 2 (eIF2α) [58,59]. Phosphorylation of eIF2α by PKR inhibits both host and viral mRNA translation, effectively suppressing viral replication [59]. The absence of PKR in the mouse cells used for our infection experiments, therefore, resulted in a substantial reduction of antiviral activity.

Since the target cells used in both assays exhibited a reduced capacity to mount antiviral defenses, our findings strongly indicate that PCMV/PRV is inherently unable to infect human or mouse cells, which are equipped with functional antiviral mechanisms. This observation is consistent with previous reports showing that PCMV/PRV does not infect human 293 or B cells [14], as well as with findings from non-human primate and human recipients of PCMV/PRV-positive xenotransplants, in whom no evidence of infection has been detected [5,36,37].

For the infection assay, a highly efficient co-incubation method was applied in which PCMV/PRV-producing cells were directly mixed with target cells resistant to a specific selection marker [60]. The resistance gene enables selective elimination of virus-producing cells upon addition of the appropriate selection medium. This assay supports both infection by cell-free virus and cell-to-cell transmission, while allowing complete removal of the virus-producing pig cells. To verify full elimination of pig cells, detection of the porcine SINE PRE1 sequence was employed as a highly sensitive confirmation method. In Sus scrofa, approximately 1.7 million SINE copies have been reported [61], of which more than one million are PRE1 elements [62]. In Chinese pig breeds, 1.98 million PRE1 copies have been detected [63]. Our finding of 10^8^ SINE PRE1 copies in the PFT cells (Table 4) falls within a comparable range. Similar to porcine SINEs, very high copy numbers have also been reported in mice, with approximately 564,000 B1 copies in the mouse genome when sequencing the genome of a female C57BL/6J mouse [64,65]. We found 10^6.9^, i.e., 7,940,000, cells in the immortalized MI4Y-3/8 cells from PKR knockout mice with a 129/Sv(ev)XC57BL/6J genetic background, indicating that the copy number may be even higher. It is well known that sequencing analyses usually underestimate the true number of repetitive sequences [66].

If PCMV/PRV does not infect non-human primate or human cells, how does it contribute to transplant dysfunction and severe recipient complications, such as disseminated microvascular hemorrhage? We previously proposed [67] that PCMV/PRV replicates efficiently in the transplanted pig heart, as reflected by the markedly higher viral copy numbers in the explanted pig heart compared with all other baboon organs [36], and by the continuously increasing viral load observed in the first patient who received a pig heart [5,41]. Viral replication within the graft likely enables viral proteins to interact directly with immune and endothelial cells, leading to elevated IL-6 and TNF-α levels, increased concentrations of tissue plasminogen activator (tPA) and tPA–plasminogen activator inhibitor-1 (PAI-1) complexes [36], and upregulation of intercellular adhesion molecule-1 (ICAM-1) and major histocompatibility complex (MHC) class II expression in the graft endothelium [34]. Such interactions are consistent with known mechanisms whereby viral proteins modulate host responses even in the absence of productive infection; for example, retroviral transmembrane envelope proteins can interact with immune cells and induce immunosuppression both in vivo and in vitro (reviewed in [68]).

## 5. Conclusions

PCMV/PRV has been transmitted in numerous preclinical xenotransplantation studies and in the first transplantation of a pig heart into a human patient, where it contributed to the death of the recipient. Here, we demonstrate that PCMV/PRV does not infect human or mouse cells. Given that these cells exhibit a markedly reduced antiviral defense capacity, it can be concluded that the virus is intrinsically unable to infect human or mouse cells, which are equipped with functional antiviral mechanisms. Consequently, PCMV/PRV likely acts indirectly on recipient immune and endothelial cells to induce pathological changes.

## Figures and Tables

**Table 2:** Detection of PCMV/PRV and PERV-C in PFT cell lines (gene copy number per 100 ng DNA in the case of PCMV/PRV and negative as tested by conventional PCR in the case of PERV-C).

Cell Line	PCMV/PRV	PERV-C	pGAPDH
PFT	n.d.	-	10^5.4^
PFT-PCMV/PRV	10^4^	-	10^5.4^

n.d., not detected.

**Table 3:** Screening for PCMV/PRV in the supernatant and in the pelleted virus fraction (gene copy number per 50 ng DNA).

Supernatant	PCMV/PRV
Direct	n.d.
Virus pellet	10^2.4^

n.d., not detected.

**Table 4:** Real-time screening for PCMV/PRV infection in co-culture experiments after 21 days (gene copy number per 100 ng DNA).

Cell Lines	PCMV PRV	PRE-1 (Porcine SINE)	SINE B1 (Murine SINE)	Human GAPDH
PFT PCMV/PRV cells	10^4^	10^8^	n.a.	n.a.
PFThyg cells after co-culture with PFT PCMV/PRV cells and subsequent selection	10^4^	10^8^	n.a.	n.a.
293Thyg cells after co-culture with PFT PCMV/PRV cells and subsequent selection	n.d.	n.d.	n.a.	10^5.2^
MI4Y-3/8 cells after co-culture with PFT PCMV/PRV cells and subsequent selection	n.d.	n.d.	10^6.9^	n.a.

n.a., not applicable; n.d., not detected. The standard curves for the determination of the copy number of PCMV/PRV [9] and porcine SINE [34] were published; the standard curve for murine SINE is shown in Figure S1.

**Table 5:** Detection of PCMV/PRV in PFT cell lines treated with infected culture supernatants after 25 days (gene copy number per 100 ng DNA).

Cell Line	PCMV/PRV	pGAPDH
PFT cells after treatment with supernatant from PCMV/PRV-producing cells	10^3.4^	10^4.5^

## Data Availability

All data are present in this manuscript.

## Supplementary Materials

The following supporting information can be downloaded at: https://www.mdpi.com/article/10.3390/v18010021/s1. Figure S1: Standard curve of the real-time PCR using primers and probes for the murine SINE B1 sequence.

## Abbreviations

The following abbreviations are used in this manuscript:

GAPDH	Glyceraldehyde 3-phosphate dehydrogenase
PERV	Porcine endogenous retrovirus
PCMV/PRV	Porcine cytomegalovirus/porcine reoseolovirus
PFT	Porcine fallopian tube
SINE	Short Interspersed Nuclear Element
SuHV-2	Suid herpesvirus 2
