# Supplementary figures and images for: Porcine Cytomegalovirus/Porcine Roseolovirus, Previously Transmitted During Xenotransplantation, Does Not Infect Human 293T and Mouse Cells with Impaired Antiviral Defense
Source: Viruses. 2025 Dec 23;18(1):21. doi: 10.3390/v18010021 (PMC12846669; doi:10.3390/v18010021)

Supplementary material, Figure S1

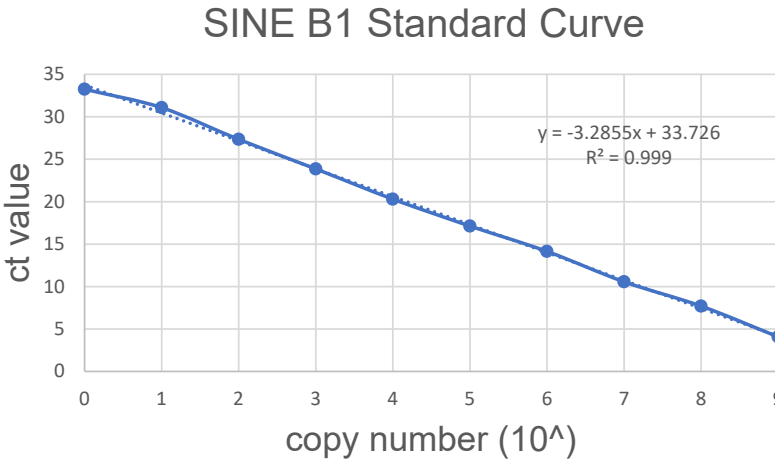

Supplement: Supplementary file 1 [file viruses-18-00021-s001.zip › viruses-4030229-supplementary.pdf]
